# Novel ST-Specific Molecular Target-Based Method for Simultaneous and Quantitative Detection of *Staphylococcus aureus* ST7, ST188 and ST398

**DOI:** 10.3390/molecules30193889

**Published:** 2025-09-26

**Authors:** Baoqing Zhou, Xiang Nie, Xudong Mao, Jiaxin Chen, Jiawen Chen, Bingfeng Ma, Xin Wu

**Affiliations:** 1 State Key Laboratory of Food Science and Resources, Nanchang University, Nanchang 330047, China; 2 Office of Science and Technology, Jiangxi General Institute of Testing and Certification, Nanchang 330052, China; 3 Nanchang Key Laboratory of Food Rapid Testing, Jiangxi General Institute of Testing and Certification, Nanchang 330200, China; 4 School of Chemistry and Biological Engineering, University of Science and Technology Beijing, Beijing 100083, China

**Keywords:** *Staphylococcus aureus* sequence types, pan-genome analysis, specific molecular target, multiplex and quantitative PCR methods, actual sample detection

## Abstract

*Staphylococcus aureus* is a globally crucial foodborne pathogen that can cause diarrhea, vomiting, and bloodstream infection in immunocompromised individuals. *S. aureus* has three predominant sequence types (STs) (ST7, ST188 and ST398) that are prevalent clones in both food and clinical cases. This study aimed to screen ST-specific targets for *S. aureus* ST7, ST188 and ST398, and then developed a novel rapid and accurate assay for the detection of these three predominant *S. aureus* STs in food. A total of 505 *Staphylococcus* strain genome sequences including 371 sequences of 58 different STs and 134 other non-target *S. aureus* ST genome sequences were subjected to pan-genome analysis; we successfully screened five novel ST-specific targets (group_10498 and group_10499 target for *S. aureus* ST7, group_9415 and group_9419 target for *S. aureus* ST188, group_9911 target for *S. aureus* ST398). The excellent specificity and sensitivity of all the targets were confirmed by PCR assays. Based on these molecular targets, mPCR and qPCR methods were developed for specifically identifying *S. aureus*’ three predominant STs without non-target bacterial interference. The limits of detection (LODs) for the mPCR assay in artificially contaminated milk were determined to be 10^4^ CFU/mL for ST7, 10^5^ CFU/mL for ST188, and 10^4^ CFU/mL for ST398, while the LODs achieved by the qPCR method were 8.6 × 10^2^ CFU/mL, 1.2 × 10^2^ CFU/mL, and 6.4 × 10^3^ CFU/mL, respectively. The testing results for actual food samples suggested that the developed mPCR or qPCR assays could be used as an alternative to standard MLST analysis, for the rapid and reliable identification of *S. aureus* STs. The novel molecular detection technology established in this study provides an efficient and reliable detection method for the prevention and control of predominant *S. aureus* ST contamination in food and has important application potential and promotion prospects.

## 1. Introduction

*Staphylococcus aureus* is a globally crucial foodborne pathogen. The toxins produced by *S. aureus* can cause diarrhea, vomiting, bloodstream infection, purulent infection, etc., all of which seriously threaten human health [1,2]. Approximately 241,000 illnesses in the United States and 20–25% of foodborne bacterial outbreaks in China have been caused by *S. aureus* contamination each year [3,4]. Moreover, the widespread use of antibiotics has increased the prevalence of multidrug-resistant *S. aureus*, bringing new challenges to clinical treatment [5]. Therefore, accurate analysis of the virulence and prevalence of *S. aureus* is significant for evaluating the potential implications of the presence of this microorganism for food safety and public health.

Currently, various molecular subtyping approaches, including pulsed-field gel electrophoresis (PFGE), staphylococcal protein A (*spa*) typing and multilocus sequence typing (MLST), have been developed for *S. aureus* characterization [6]. However, PFGE analysis is relatively complicated, and the same PFGE model often shows different biochemical characteristics, which has limited its application in subtype analysis [7]. *spa* typing based on single locus analysis would limit the resolution for epidemiological typing, and recombination events might distort the underlying clonal relationships [8]. These factors have restricted their applications in practical use in the food industry and clinical application. As a robust technique, MLST, which is based on sequences of single-nucleotide polymorphisms from seven housekeeping genes to classify sequence types (STs) and clonal complexes (CCs), has proven useful for both epidemiology and evolutionary studies [9]. Studies tracking outbreaks related to *S. aureus* have revealed differences in pathogenicity at the intra-species level that are closely associated with specific *S. aureus* STs [10]. For example, Song et al. (2016) [11] found that the *S. aureus* ST188 strains were closely associated with outbreaks of staphylococcal food poisoning (SFP). Some studies reported that, in atopic dermatitis and pediatric patients with bloodstream infections, predominant methicillin-susceptible *S. aureus* (MSSA) clones belonged to ST188 and ST7 types [12,13]. A recent study revealed that ST7 became one of the most common *S. aureus* STs (the MRSA proportion of ST7 increased from 19.1% to 50%) after the COVID-19 epidemic in the city of Wuhan, China [14], and ST7 was the main reported ST of *S. aureus* in local wholesale and retail pork (detection rate: 57.5%) [15]. ST7, ST188 and ST398 clones were found to be multidrug-resistant, have strong biofilm formation ability and have a higher positive rate of a variety of virulence genes [14,15,16]. Relevant studies have shown that livestock-associated methicillin-resistant *S. aureus* (LA-MRSA) ST398 strains are prevalent in livestock farms, communities and hospitals in Europe and North America, while methicillin-susceptible *S. aureus* (MSSA) ST7, ST188 and ST398 are predominant in Asia [12,17,18,19,20]. Moreover, our previous work has proved that the *S. aureus* ST7, ST188 and ST398 strains are the predominant and persistent pathogens in the contamination of fresh rice and flour products, retail meat products and pasteurized milk [21,22]. Therefore, the accurate identification of these three predominant *S. aureus* STs’ occurrence and contamination is of utmost significance for food safety and clinical diagnosis.

According to the Industry Standard of SN/T 4525.2-2016 (MLST-based molecular typing method of Pathogenic Bacteria in Exported Food, Part 2: *Staphylococcus aureus*), the predominant steps for *S. aureus* ST identification include genomic DNA extraction, housekeeping gene amplification, product purification and sequencing analysis, data upload, and sequence alignment, which are expensive, time-consuming and labor-intensive [23]. MLST analysis of *S. aureus* can also be completed by online analysis (https://pubmlst.org/databases/, accessed on 20 May 2025) with whole genome data using high-throughput sequencing [24]. Nevertheless, high accuracy and great specificity of this method can hardly conceal its shortcomings as costly and time-consuming. To overcome these shortcomings, PCR-based molecular methods have been developed as promising alternatives for the traditional *S. aureus* MLST analysis. Notably, the selection of highly specific detection targets directly determines the sensitivity and accuracy of molecular detection methods. However, a limited number of specific targets for MLST analysis have been reported so far, such as targets *sau1-hsdS1*, *A07*, and *C01* for identification of *S. aureus* ST398 [25,26,27] and targets *SA0317* and *SA2003* for *S. aureus* ST239 [28], but there were no targets for ST7 and ST188 as well as other ST strains. With the increase in available *S. aureus* genomic sequencing data and new isolated *S. aureus* STs, some reported molecular targets regarded as ST-specific may inevitably be isolated in other STs, and they also have limitations in accuracy and specificity. Therefore, mining of novel ST-specific molecular targets and development of rapid and quantitative technology are essential for accurate identification of the *S. aureus* predominant STs.

**Figure 1:**
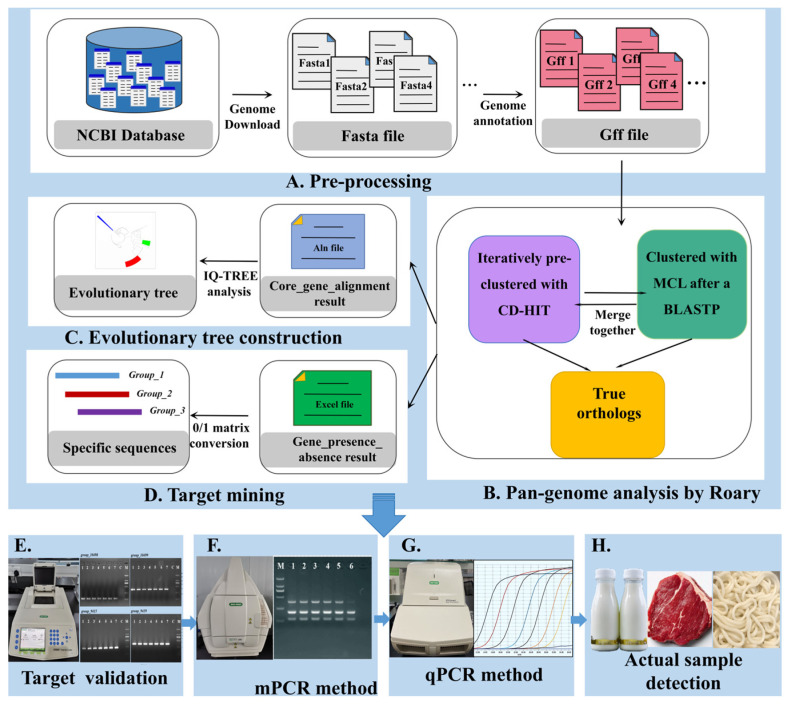
The workflow for establishment of special target screening and molecular methods for predominant *S. aureus* STs. The workflow of *S. aureus* evolutionary tree construction (A → B → C). The workflow for special target screening of *S. aureus* STs (A → B → D). Establishment of molecular target-based methods for detection of STs (D → E → F → G → H).

The pan-genome is composed of core and auxiliary gene banks and is a useful framework for describing the diversity of genomes in taxa. Based on the whole-genome data, pan-genomic analysis has great potential in the field of target mining. At present, a large number of *S. aureus* strains have been fully sequenced (NCBI bank, https://www.ncbi.nlm.nih.gov/, accessed on 10 January 2025), which provided sufficient information on the diversity of *S. aureus* STs. Using pan-genomic analysis, the potential molecular targets specific for different *S. aureus* STs can be easily mined. Thus, the aims of this study were to (1) establish a target screening platform based on pan-genome analysis to mine the novel ST-specific targets for detection of the three predominant *S. aureus* STs; and (2) establish mPCR and qPCR methods using novel molecular targets for simultaneous and quantitative detection of predominant *S. aureus* STs in actual samples (Figure 1).

## 2. Results

### 2.1. Phylogenetic Analysis of S. aureus Isolates

A core genome was determined for each isolate using a 99% cutoff [29]. We identified 1258 core genomes among the selected 371 *S. aureus* isolates through pan-genome analysis, and then constructed a phylogenetic tree using IQ-TREE 2.0 software.

As shown in Figure S1, isolates were distributed across different STs, and most isolates belonged to ST7, ST188 and ST398. Notably, isolates belonging to different STs were highly discriminated, suggesting an evolutionary divergence between STs.

### 2.2. Identification of ST-Specific Genes of S. aureus

To screen specific candidate targets for the detection of the three predominant STs of *S. aureus*, we determined the distribution and size of the *S. aureus* pan-genome across the 505 genomes with a BLASTP identity cutoff of 85% [29]. A total of five specific markers were identified for these three STs. Notably, two (*group_10498* and *group_10499*), two (*group_9419* and *group_9415*), and one (*group_9911*) specific gene marker were found in ST7, ST188 and ST398, respectively, and coded for unknown proteins. Whereas *A07*, *C01* and *sau1-hsdS1* were previously reported as markers of ST398 [25,26,27], there were no targets reported for the detection of ST7 and ST188. The percentages of genomic sequences harboring these genes are displayed in Table 1. Compared with the reported targets, for both the targeted and non-targeted STs, the specific genes in our study displayed better performance in specificity with the same positive coverage.

### 2.3. Evaluating Specificity and Sensitivity of ST-Specific Genes Using PCR Assay

Primer sequences designed based on the ST-specific genes are listed in Table 2. Specificity of these primer pairs was determined by PCR using the related strains (Table 3). As expected, all primer sets designed on ST7, ST188 and ST398 specific genes could amplify target bands corresponding to the target strains, while other non-target strains produced no bands (Figure S2), which validated the excellent specificity of these primers. Then, for the anti-interference test of these primers, the other DNA samples from *S. aureus* ST8 1-1 of 10^1^~10^7^ CFU/mL were mixed with the three predominant *S. aureus* STs (initial concentration of 10^6^ CFU/mL), respectively. As shown in Figure S3, all amplicons generated by target strains with other bacterial interference showed uniform and clear bands, indicating that all novel primers had excellent anti-interference abilities.

The sensitivity of identification for target genes specific to ST7, ST188 and ST398 was further verified. The LODs for group_10498 and group_10499 (specific for *S. aureus* ST7) were 8.6 × 10^3^ CFU/mL and 8.6 × 10^4^ CFU/mL, respectively, those for group_9419 and group_9415 were 1.2 × 10^4^ CFU/mL and 1.2 × 10^3^ CFU/mL (specific for *S. aureus* ST188), respectively, and for group_9911, 6.4 × 10^4^ CFU/mL (specific for *S. aureus* ST398) (Figure S4).

### 2.4. Detection of Predominant S. aureus STs in Artificially Contaminated Milk

To evaluate the suitability of ST-specific gene-based PCR methods, we firstly established an mPCR assay for the simultaneous identification of three *S. aureus* STs in spiked milk. Based on the principle of high primer amplification efficiency and appropriate product length matching, three primer sets from the target genes (*group_10498*, *group_9419* and *group_9911*) were selected for mPCR analysis.

**Figure 2:**
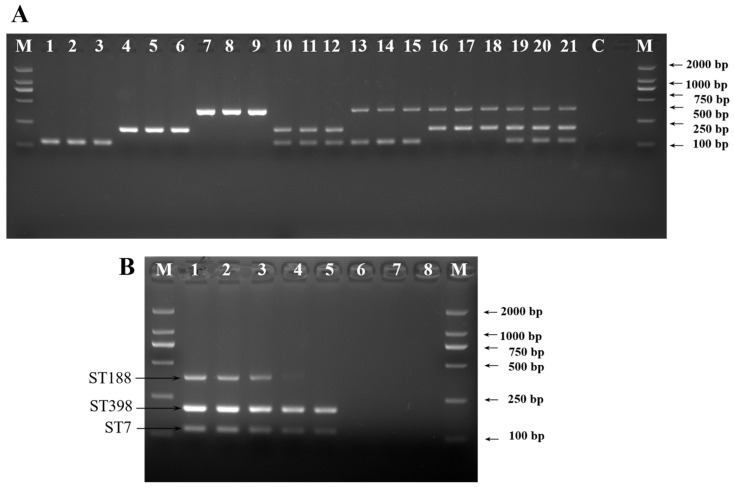
The mPCR results for detection of predominant *S. aureus* STs in artificially contaminated milk. (**A**) Feasibility analysis of mPCR method, lanes 1–3: target ST7, lanes 4–6: target ST398, lanes 7–9: target ST188, lanes 10–12: ST7 + ST398, lanes 13–15: ST7 + ST188, lanes 16–18: ST188 + ST398, lanes 19–21: ST7 + ST188 + ST398. (**B**) Sensitivity of mPCR for simultaneous detection of predominant *S. aureus* STs, lanes 1–8: Mixture of predominant *S. aureus* STs’ template DNA from 10^8^ to 10^1^ CFU/mL. M: DL2000 DNA marker, C: negative control.

Under optimal conditions, the mPCR assays were successful in clearly identifying the three target STs (Figure 2A). To assess the sensitivity of the mPCR assay, different concentrations of genomic DNA were tested. Three clear bands for all three target genes in lane 4 and two bands for *group_10498* and *group_9911* in lane 5 were observed, as shown in Figure 2B, determining that the LODs of mPCR for detection of ST7, ST188 and ST398 were 8.6 × 10^4^ CFU/mL, 1.2 × 10^5^ CFU/mL, and 6.4 × 10^4^ CFU/mL, respectively.

**Figure 3:**
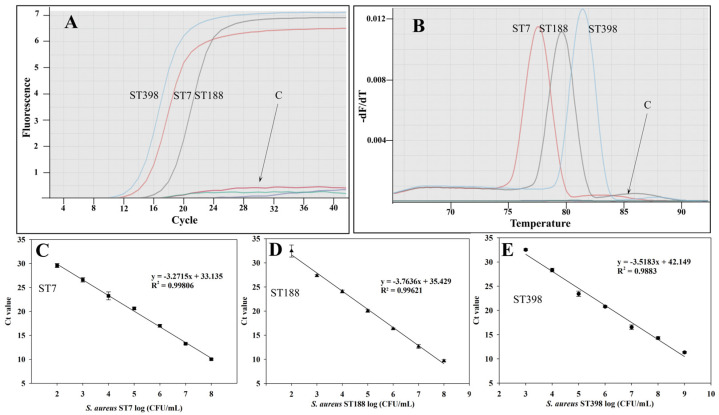
The qPCR results for quantitative detection of predominant *S. aureus* STs in artificially contaminated milk. (**A**) Amplification curve analysis of qPCR methods. (**B**) Resolution melting analysis of qPCR methods; (**C**–**E**) Establishment of standard curves by cycle threshold (Ct) values against the log numbers of cells of three *S. aureus* STs, *S. aureus* ST7, ST188 and ST398, in a range of 10^3^~10^9^ CFU/mL, respectively. All results were collected in triplicate.

For quantitative detection, we developed a qPCR assay for real-time testing on the three *S. aureus* STs. As shown in Figure 3A,B, the typical amplification curve and sharp melting curve of target genes indicated this qPCR assay could successfully quantify the three *S. aureus* STs. The LODs for the quantitative determination of ST7, ST188 and ST398 were 8.6 × 10^2^ CFU/mL, 1.2 × 10^2^ CFU/mL, and 6.4 × 10^3^ CFU/mL, respectively (Figure 3C–E).

To determine the specificity of the mPCR and qPCR methods, genomic DNA from 30 target *S. aureus* ST strains and 30 non-target *S. aureus* ST strains were extracted. As shown in Table S1, regardless of the mPCR or qPCR method, positive results were obtained for all target *S. aureus* ST strains, while negative signals were obtained for non-target bacterial strains, indicating excellent specificity for identification of the three *S. aureus* STs.

### 2.5. Anti-Interference Test for mPCR and qPCR

**Figure 4:**
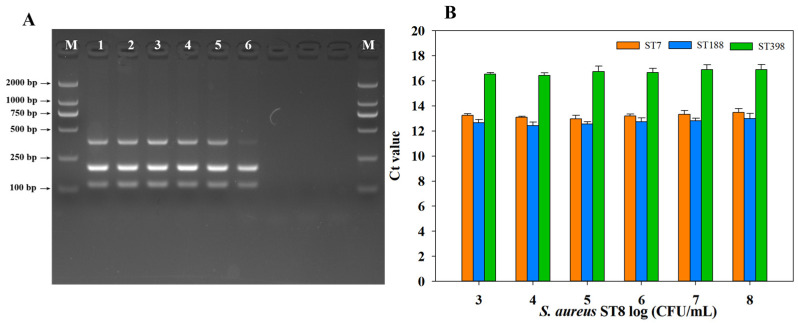
Anti-interference evaluation of mPCR (**A**) and qPCR (**B**) assays for detection of three predominant *S. aureus* STs. Lanes 1–6: non-target *S. aureus* ST8 of 10^3^~10^8^ CFU/mL mixed with three predominant *S. aureus* STs (initial concentration of 10^7^ CFU/mL), respectively, M: DL2000 DNA marker, Ct values: cycle threshold (Ct) values.

As shown in Figure 4A, a faint band was observed in the mPCR detection of *S. aureus* ST188 only when the concentration of *S. aureus* ST8 reached 10^8^ CFU/mL. For samples interfered with by non-target bacteria at other concentrations, correct and clear bands could be produced using the mPCR method. As shown in Figure 4B, no significant differences were noted in the amplification cycle threshold (Ct) values of the qPCR assay for detection of the three target *S. aureus* STs across different concentrations of interfering non-target *S. aureus* ST8. These results demonstrate that although the detection signals of the mPCR and qPCR methods established in this study are subject to certain interference under different concentrations of non-target *S. aureus* ST8, it is acceptable that these methods have strong anti-interference ability and adaptability.

### 2.6. Application of mPCR and qPCR in Natural Milk to Detect Three S. aureus STs

All detection results for the mPCR and qPCR assays are listed in Table 4. When positive control samples gave accurate results, only one pork meat and one wet rice noodle sample tested positive for *S. aureus* ST188, and none of the samples tested positive for *S. aureus* ST7 or ST398 by mPCR or qPCR assay.

As shown in Table 5, the corresponding sensitivity, specificity, and efficiency values were all 100% for the identification of the three predominant *S. aureus* STs, indicating that the established mPCR and qPCR methods maintained good consistency with the standard MLST method for detection of the three predominant *S. aureus* STs.

## 3. Discussion

Molecular subtyping is the most commonly used assay for phenotypic characterization for identification of *S. aureus* isolates. Molecular subtyping could rapidly determine the association between virulence and source of *S. aureus*, which is useful in long-term epidemiology and evolutionary studies. As the most commonly used molecular subtyping method for *S. aureus* characterization, MLST is based on sequences of single nucleotide polymorphisms from seven housekeeping genes to classify STs and CCs, but it is too time-consuming and labor-intensive for the needs of large-scale screening [30]. The molecular target-based PCR method provides an alternative approach for the detection of target *S. aureus* STs [2]. However, there are few available ST-specific targets for *S. aureus* identification, except *sau1-hsdS1*, *A07*, and *C01* used for identification of ST398 [25,26,27]. Targets *SA0317* and *SA2003* were simultaneously used to identify ST239 [28], and the mPCR method was used for ST764 identification [31]. Moreover, reported targets for detection of different STs are still limited in accuracy and specificity, because similar sequences are screened in different STs. Thus, there is a definite need for mining novel ST-specific molecular targets for reliable detection of other predominant *S. aureus* STs.

In this work, we screened novel ST-specific targets using a pan-genome analysis on 371 *S. aureus* genome sequences belonging to 58 different STs and 134 other sequences belonging to other species of *Staphylococcus*. The pan-genome analysis revealed 27,961 protein-coding genes, including 939 core genes. Isolates belonging to different STs were highly discriminated, suggesting an evolutionary divergence between STs. Five novel ST-specific molecular targets were screened. Among them, the specific targets for ST7 and ST188 identification have not been reported before. The discovery of novel ST-specific molecular targets provides a low-cost, more efficient, and convenient approach for the rapid MLST of *S. aureus*. Notably, all five target genes encoded hypothetical proteins with little or no information on their function. The identification of these specific hypothetical protein-coding regions can not only improve the accuracy of our established rapid detection methods but also contribute to the analysis of gene structures and functional roles and ultimately promote in-depth understanding of the unique metabolic behaviors and associations of different *S. aureus* STs [32]. We will conduct more experiments to verify the functions of key unknown target genes. For instance, ref. [33] constructed knockout mutants of unknown genes in target strains and evaluated changes in their phenotypic characteristics (such as biofilm-forming ability and pathogenicity in animal models), and investigated the expression levels of unknown genes under various stress conditions, among other related work. More in-depth functional annotation would lay a solid foundation for exploring the biological significance of these targets in the pathogenic process of *S. aureus*.

The usefulness of a PCR-based assay is largely dependent on the specificity and sensitivity of the target sequence. Previous work for the identification of specific targets was focused on relatively small genomic datasets including gene fragments or virulence genes. For example, van Belkum et al. (2008) [25] screened the ST398-specific targets *SAPIG2194* (*A07*) and *SAPIG2195* (*C01*) by amplified fragment length polymorphism (AFLP) analysis based on 147 marker fragments from 46 pig-related MRSA isolates. Stegger et al. (2011) [28] identified the ST398-specific target region (530 bp) in *sau1-hsdS1 by* aligning seven of the publicly available *sau1-hsdS1* sequences. According to the reported results, the specificity of the reported targets for ST398 detection could be limited by genetic mutations [34]. In contrast to previous target mining based on coding sequences or single virulence genes, we carried out a pan-genome analysis approach to screen ST-specific targets with high reliability. Pan-genome analysis, which is based on whole-genome sequence alignment, has been used to mine specific markers in bacteria, such as virulence, serovar, molecular subtyping and antibiotic resistance. All markers are usually related to genes acquired though horizontal transfer from the genes of other species, and usually present in species showing corresponding phenotypes [35,36]. Undoubtedly, this study has only established a rapid detection technology for three important *S. aureus* STs, which is insufficient to meet practical detection needs. In subsequent work, we will further improve the mining of ST molecular targets to achieve accurate identification of other *S. aureus* STs.

Due to the expansion of the genome database, some nucleotide targets previously considered to be specific are inevitably eliminated. To obtain truly reliable and specific targets, a combination between bioinformatics analysis and a PCR verification based on an extensive collection of bacterial strains is crucial. As shown in Table 1, the novel screened ST-specific targets in this work exhibited better performance compared with those in previously reported studies, and all reported targets showed a slight overlap with the genomes of other species, while the percentages of genomic sequences present in target species and non-target strains were 100% and 0%, respectively. Moreover, we evaluated the ST398-specific targets *A07* and *C01* using 81 target bacterial strains and 71 other bacterial strains, and found two target genes also matched with genomes of other species, particularly target *C01* (Figure S5), which was consistent with the reported results [34]. In comparison, molecular targets mined in our work were more specific to three predominant *S. aureus* STs. In addition, specificity verification results using 81 target bacterial strains and 71 other bacterial strains also showed excellent performance.

In order to rapidly identify *S. aureus* STs, some ST-specific target-based PCR methods have been applied [25,26,27,28,31]. However, these methods often require combining multiple targets to distinguish specific STs, which is not conducive to the rapid detection of *S. aureus* STs. In this study, we achieved simple detection of *S. aureus* STs based on single specific targets. To further achieve simultaneous and quantitative identification of target bacteria, we firstly established an mPCR method based on the novel targets to detect three predominant *S. aureus* STs, which showed excellent performance in specificity and anti-interference ability. Then, a qPCR assay was developed for real-time identification of target bacteria. The LODs for quantitative determination of *S. aureus* ST7, ST188 and ST398 in spiked milk were 8.6 × 10^2^ CFU/mL, 1.2 × 10^2^ CFU/mL, and 6.4 × 10^3^ CFU/mL respectively. The developed qPCR assay has a lower cost and better sensitivity than that reported in other work [28,37]. The mPCR method established in this study has the advantages of low cost and simple operation and can simultaneously detect three *S. aureus* STs, making it particularly suitable for rapid screening scenarios in primary-level institutions. In comparison, the qPCR method is significantly superior to the mPCR method in terms of detection sensitivity and anti-interference capability. It can achieve accurate quantification of a single target bacterium and provide precise data support for clinical diagnosis and treatment. However, the mPCR and qPCR technologies established in this study would have difficulty meeting the POCT requirements, and false positive results may occur due to aerosol contamination during the detection process. Zhang et al. (2025) [38] developed a recombinase polymerase amplification system combined with lateral flow immunoassay for detection of S. aureus without aerosol contamination. Savas et al. (2025) [39] established a novel smartphone-based nanozyme-enhanced electrochemical immunosensor for *S. aureus* POCT. The developed biosensor effectively detected *S. aureus*, with a calculated LOD of 4 CFU/mL in undiluted milk. Combining isothermal amplification technology and test strip technology can effectively improve the method established in this study, thereby truly achieving accurate identification of three *S. aureus* STs. We successfully applied the mPCR assay and qPCR method to identify *S. aureus* ST7, ST188 and ST398 in natural food samples. Compared with the standard MLST analysis, our assay was more rapid and convenient [40], providing a preliminary foundation for its use as an alternative solution to the traditional MLST method.

## 4. Material and Methods

### 4.1. Bacterial Strains and Genomic DNA Extraction

A total of 198 strains, including 172 *S. aureus* strains representing 48 different STs and 26 non-*S. aureus* strains were used in this study (Table 3). These reference strains were isolated from our laboratory, while a few standard strains obtained from American Type Culture Collection (ATCC, Manassas, VA, USA) and China Medical Culture Collection (CMCC, Beijing, China). All test bacteria were grown in Luria-Bertani (LB) medium (Guangdong Huankai Co., Ltd., Guangzhou, China) at 37 °C. And the genomic DNA from overnight cultures was extracted with bacterial DNA extraction Mini Kit (Mabio, Guangzhou, China). A microplate reader (Epoch 2, BioTek Instruments Inc., Winooski, VT, USA) and Qubit^®^ 3.0 Fluorometer (Life Invitrogen, Waltham, MA, USA) were used to determine the purity and concentration of genomic DNA respectively, and the extracted DNA was stored at −20 °C before use.

### 4.2. Phylogenetic Analysis

As shown in Figure 1 (A→B→C), all analyzed *S. aureus* genome sequences downloaded from NCBI were annotated with Prokka v1.11 [41]. The annotated files (.gff) were collected to perform pan-genome analysis using Roary v3.11.2 [42]. The obtained data (.aln) of a core genome alignment of *S. aureus* were then used for efficient tree construction using IQ-TREE software 2.0 on a linux platform [43], and the final result was then visualized using iTOL v7.2.1 (https://itol.embl.de/upload.cgi, accessed on 20 May 2025).

### 4.3. Screening of S. aureus ST-Specific Targets

The 505 *Staphylococcus* strain genome sequences contained 371 *S. aureus* sequences of 58 different STs and 134 other non-target *S. aureus* STs, and the corresponding sequence information is shown in Table S2. The ST-specific molecular targets were screened through pan-genome analysis using Roary software; the workflow is shown in Figure 1 (A → B → D). After the pan-genome analysis, the existence/non-existence profile of all genes across strains was determined and then converted into a 0/1 matrix using a local script. *S. aureus* FORC59, *S. aureus* 08-02300 and *S. aureus* subsp. aureus ST398 were selected as the references for target identification. The screening criteria were as follows: 100% presence in target *S. aureus* ST strains and 0% presence in non-target *S. aureus* ST strains. The candidate ST-specific genes were then aligned using the BLAST 2.17.0 program to further validate their specificity.

### 4.4. Primer-Based Evaluation of Novel ST-Specific Targets

Five candidate targets specific for the *S. aureus* STs were screened by pan-genome analysis. The primer pairs for these *S. aureus* ST-specific targets were designed using Primer Premier 6.0 software and synthesized by Sangon Biotech Co., Ltd. (Shanghai, China) (Table 2).

The PCR amplifications were performed in a 10 μL PCR reaction volume including 1× Taq Master (Dongsheng, Guangzhou, China), 5 μM of primer pairs, 1 μL of genomic DNA and 3 μL ultrapure water using a Biometra TOne 96G thermal cycler (Analytik Jena, Jena, Germany). The cycling conditions were as follows: initial denaturation at 95 °C for 10 min, followed by 30 cycles of denaturation at 95 °C for 30 s, 57 °C for 40 s, and 72 °C for 30 s, and a final extension at 72 °C for 10 min. The amplicons were run on a 1.5% agarose gel electrophoresis followed by a visualized detection using an automatic digital gel image analysis system (Tanon-2500; Tanon Science & Technology Co., Ltd., Shanghai, China). The specificity of each primer pair was determined against the bacterial strains listed in Table 2. Limits of detection (LODs) were determined with varied concentrations of genomic DNA extracted from fresh cultures of the *S. aureus* 151-0 strain (ST7), *S. aureus* 126-0 strain (ST188), and *S. aureus* 322-1 strain (ST398), respectively.

### 4.5. Multiplex PCR and Quantitative PCR Conditions

For the simultaneous detection of three *S. aureus* STs (ST7, ST188 and ST398), mPCR assays based on three specific primers were performed, and contained 12.5 μL of PCR mix, 0.2 μM of primers targeting *S. aureus* ST188 and *S. aureus* ST398, 0.5 μM of primers targeting *S. aureus* ST7, and 1 μL each of the three template DNAs, as well as ultrapure water added to achieve a 25 μL volume. The cycling program was as follows: initial denaturation at 95 °C for 10 min, followed by 30 cycles of denaturation at 95 °C for 30 s, 57 °C for 40 s, and 72 °C for 30 s, and a final extension at 72 °C for 10 min. A 1.5% agarose gel electrophoresis was used to separate all amplified products.

For the quantitative detection of these predominant *S. aureus* STs, real-time PCR assays based on three specific primers were also performed. Three independent dye-based qPCR methods were prepared, and each reaction mixture contained 5 μL of TB Green™ Premix Ex Taq™ II (TaKaRa, Biotech, Dalian, China), 0.5 μM of primer pairs and 1 μL template DNA, as well as ultrapure water added to reach a 10 μL volume. The qPCR was performed on a Light Cycler^®^ 96 real-time PCR system (Roche, Basel, Switzerland), and the thermal cycling was as follows: denaturation at 95 °C for 100 s, followed by 45 cycles of denaturation at 95 °C for 10 s and annealing at 60 °C for 30 s. The data were analyzed using LightCycler® 96 1.1.0.1320 software. All results were collected in triplicate.

### 4.6. Sensitivity of the mPCR/qPCR Assays in Artificially Contaminated Milk Samples

For the LOD evaluation, 25 mL of milk determined to be negative for *S. aureus* by standard culture methods was homogenized in 225 mL of sterile saline solution. Fresh cultures with strains of these three *S. aureus* STs were mixed with homogenate to prepare varied concentrations of substrate ranging from 10^1^~10^8^ CFU/mL, then all of the genomic DNA was extracted for mPCR and qPCR analysis.

### 4.7. Evaluation of Specificity and Anti-Interference Capability of mPCR/qPCR Assays

The specificity of the mPCR and qPCR assays was determined using 30 target *S. aureus* ST strains and 30 non-target *S. aureus* ST strains (Table S1), and ultrapure water instead of genomic DNA was used as a negative control. As specificity criteria, primer sets that amplified target bands or generated obvious fluorescence signals from the corresponding target ST strains but not from non-target *S. aureus* ST strains were considered specific.

To evaluate the accuracy of the mPCR and qPCR methods under the interference of contaminant bacteria, fresh cultures of the three target *S. aureus* ST strains were mixed with *S. aureus* strain 1-1 (ST8) at different concentrations, resulting in final concentration ratios of 10^7^:10^8^, 10^7^:10^7^, 10^7^:10^6^, 10^7^:10^5^, 10^7^:10^4^, and 10^7^:10^3^, respectively. DNA templates from all samples were extracted, and detection was performed using the mPCR and qPCR methods, with each sample tested in triplicate.

### 4.8. Application of the mPCR and qPCR Assays for the Analysis of Food Samples

To determine the validity of the mPCR/qPCR assays for *S. aureus* ST identification in food samples, a comparative study was performed using the gold standard method (according to guidelines from National Food Safety Standards of China GB 4789.10-2016). Briefly, a total of 30 food samples (including 10 milk, 10 pork meat and 10 wet rice noodle samples) were purchased from local markets, and 25 g/25 mL of each sample was homogenized in 225 mL of sodium chloride broth (Huankai, Guangzhou, China), followed by thorough mixing and culturing at 36 ± 1 °C for 18–24 h. Then, the enrichment cultures were streaked into Baird–Parker plates and blood plates for isolation and purification. Then, genomic DNA of an unknown strain was extracted and used as a template for mPCR and qPCR detection. In parallel, the isolate strains were identified using a combination identification of staining microscopy, hemolysis and coagulase test. The confirmed *S. aureus* isolates were then subjected to an MLST analysis according to the Industry Standard (SN/T 4525.2-2016). The performance of the qPCR and mPCR methods was further evaluated by using a correlation analysis with the standard MLST method. The main parameters included SE (sensitivity), i.e., the ability to detect positive samples; SP (specificity), i.e., the ability to detect negative samples; and the efficiency (EF); the calculation formulas are as follows:

SE (%) = a/(a + b) × 100(1)

SP (%) = c/(c + d) × 100(2)

EF (%) = (a + c)/(a + b + c + d) × 100(3)

where a is the number of true-positive samples, with positive results obtained using mPCR/qPCR and the traditional MLST method; b is the number of false-positive samples, with a negative result for the traditional MLST method and a positive result for the mPCR/qPCR methods; c is the number of true-negative samples, with negative results determined using mPCR/qPCR and the traditional MLST method; and d is the number of false-negative samples, with a positive result for the traditional MLST method and a negative result for mPCR/qPCR assay.

## 5. Conclusions

In summary, novel mPCR and qPCR methods were developed to simultaneously and quantitative detect three predominant STs of *S. aureus* in food using pan-genome analysis. Compared with the standard MLST analysis, the developed molecular methods are low in cost and easy to operate, enabling sensitive and specific detection of three predominant *S. aureus* STs. The LODs for the mPCR assay in artificially contaminated milk were determined to be 10^4^ CFU/mL for ST7, 10^5^ CFU/mL for ST188, and 10^4^ CFU/mL for ST398, while the LODs achieved by the qPCR method were 8.6 × 10^2^ CFU/mL, 1.2 × 10^2^ CFU/mL, and 6.4 × 10^3^ CFU/mL respectively. The current results indicate that the newly developed mPCR and qPCR detection methods have application potential in the detection of *S. aureus* in food, providing a preliminary foundation for subsequent exploration of their use as alternative solutions to the traditional MLST method.

## Figures and Tables

**Table 1:** Presence profile of novel and reported *S. aureus* ST-specific genes for target and non-target strains.

Target	Target Genes	Presence Profile in		Source
		Target Strain	Non-Target Strain	
*S. aureus* ST7	*group_10498*	10 (100%)	0 (0%)	This study
	*group_10499*	10 (100%)	0 (0%)	This study
*S. aureus* ST188	*group_9419*	3 (100%)	0 (0%)	This study
	*group_9415*	3 (100%)	0 (0%)	This study
*S. aureus* ST398	*group_9911*	34 (100%)	0 (0%)	This study
	*A07*	34 (100%)	2 (0.4%)	[26]
	*C01*	34 (100%)	21 (4.2%)	[26]
	*sau1-hsdS1*	34 (100%)	18 (3.6%)	[28]

**Table 2:** ST-specific target information, primer set sequences and PCR detection sensitivity results.

Target	Name of Target Gene	Encoded Protein	* Gene Location	Primer Set Name	Sequence (5′-3′)	Product Size (bp)	LOD in Pure Culture (cfu/mL)
*S. aureus* ST7	*group_10498*	hypothetical protein	2366410~2366754	ST-1 (F)	GTTACATCAGATCAAGCAGAG	119	8.6 × 10^3^
				ST-1 (R)	GCATTTAGAAAAGCAGTGG		
	*group_10499*	hypothetical protein	2366764~2367651	ST-2 (F)	CGACTATCAGTTTTACAATCC	369	8.6 × 10^4^
				ST-2 (R)	CGTATAGACCTAACCCAGC		
*S. aureus* ST188	*group_9419*	hypothetical protein	2475310~2475726	ST-3 (F)	GATGTTATTCCTATCGCAACG	238	1.2 × 10^4^
				ST-3 (R)	GAACGCCACTACTTTCACTTT		
	*group_9415*	hypothetical protein	2445882~2446304	ST-4 (F)	GCCCTATAACTTTACGACGCAG	388	1.2 × 10^3^
				ST-4 (R)	CCAACTATTGATTTGATTTACCACG		
*S. aureus* ST398	*group_9911*	hypothetical protein	2275068~2275370	ST-5 (F)	CTTCTACGATGCCTTAGC	231	6.4 × 10^4^
				ST-5 (R)	TGTTCAATGACGGTTTCT		

* Reference strains are *S. aureus* FORC59, *S. aureus* 08-02300 and *S. aureus* subsp. aureus ST398.

**Table 3:** Bacterial strains used in this study and specificity results for target primers used in PCR amplification.

No.	Bacterial Species	Strains	ST Type	Number of Strains	Source *	Special Target for PCR Results		
						ST7	ST188	ST398
1–23	*S. aureus*	7-1, 22-0, 22-1, 42-0, 42-1, 42-2, 44-1, 151-0, 151-1, 173-0, 177-0, 178-1, 178-2, 192-0, 192-1, 201-0, 201-1, 201-2, 202-1, 203-0, 203-2, 306-1, 322-1	7	23	a	+	−	−
1–23		65-1, 65-2, 126-0, 126-1, 153-0, 1475-1, 1863-1, 510A-1, 545-1, 636-1, 663-1, 742-1, 3055-1, 3151-1, 3151C1, 3153-1, 3153A1, 3153A2, 3153B3, 3185-1, 3188-1, 3231-1, 3260-1	188	23	a	−	+	−
1–81		9-0, 67-0, 229-0, 436, 489, 531, 548, 549, 646, 706, 976, 1003, 1023, 1142, 1198, 1255, 1272, 1352B, 1387-1C, 1492, 1494, 1723, 1772, 1823-0, 1879, 1929-0, 1973-0, 1973-1, 2011-1, 2092-0, 2094-0, 2094-1, 2152-0, 2155-0, 2180-0, 2183-0, 2194-1, 2197-0, 2429-1, 2517-0, 2517-1, 2553-0, 2553-2, 2566-1, 2566-2, 2651-0, 2651-1, 2680-0, 2831, 3026, 3122, 3152, 3224, 3373, 3375, 3677, 3678, 3728A1, 3755, 3755A1, 3838B1, 3838C2, 3981, 3988, 3993, 3993A1, 4022C2, 4051, 4051A3, 4076A1, 4123, 4173, 4174, 4260A1, 4266C1, 4275, 4291A1, 697A, 2816-5, 2816-8, 2831-3	398	81	a	−	−	+
1		16-0	1	1	a	−	−	−
2		396	10	1	a	−	−	−
3		922-0	1085	1	a	−	−	−
4		1393	12	1	a	−	−	−
5		2753-2	1301	1	a	−	−	−
6		3895	133	1	a	−	−	−
7		17	15	1	a	−	−	−
8		41421	1635	1	a	−	−	−
9		3456B1	1659	1	a	−	−	−
10		2874B1	1920	1	a	−	−	−
11		1843	20	1	a	−	−	−
12		3098	22	1	a	−	−	−
13		486	25	1	a	−	−	−
14		260	2592	1	a	−	−	−
15		1025	2990	1	a	−	−	−
16		353	30	1	a	−	−	−
17		1148	3055	1	a	−	−	−
18		421	333	1	a	−	−	−
19		3652C1	3355	1	a	−	−	−
20		2194-2	3685	1	a	−	−	−
21		3043	403	1	a	−	−	−
22		157-0	4062	1	a	−	−	−
23		3929	4691	1	a	−	−	−
24		2630-1	4692	1	a	−	−	−
25		3704	4693	1	a	−	−	−
26		3929	4694	1	a	−	−	−
27		4029C2	5	1	a	−	−	−
28		29-0	504	1	a	−	−	−
29		52-0	522	1	a	−	−	−
30		1022	537	1	a	−	−	−
31		1831-0	573	1	a	−	−	−
32		368	59	1	a	−	−	−
33		24-0	630	1	a	−	−	−
34		1813-0	672	1	a	−	−	−
35		2039-0	692	1	a	−	−	−
36		24-2	72	1	a	−	−	−
37		1-1	8	1	a	−	−	−
38		631	88	1	a	−	−	−
39		1-0	9	1	a	−	−	−
40		223-2	906	1	a	−	−	−
41		3251B1	943	1	a	−	−	−
42		73-1	944	1	a	−	−	−
43		3675C1	950	1	a	−	−	−
44		1190	965	1	a	−	−	−
45		707	97	1	a	−	−	−
46	*S. epidermidis*	612-1	/	1	a	−	−	−
47	*S. hominis*	0651-3	/	1	a	−	−	−
48	*S. haemolyticus*	0770-1	/	1	a	−	−	−
49	*S. capitis*	0640-3	/	1	a	−	−	−
50	*S. warneri*	0629-1	/	1	a	−	−	−
51	*S. saprophyticus*	1045-1	/	1	a	−	−	−
52	*S. sciuri*	0729-7	/	1	a	−	−	−
53	*S. lugdunensis*	0791-2	/	1	a	−	−	−
54	*S. cohnii*	0616-5	/	1	a	−	−	−
55	*S. pasteuri*	0821-1	/	1	a	−	−	−
56	*S. gallinarum*	2483-1	/	1	a	−	−	−
57	*S. hyicus*	0747-6	/	1	a	−	−	−
58	*S. equorum*	1217-4	/	1	a	−	−	−
59	*S. schleiferi*	2926B2-2	/	1	a	−	−	−
60	*S. succinus*	1580-1	/	1	a	−	−	−
61	*S. lentus*	1091-2	/	1	a	−	−	−
62	*Shigella sonnei*	0639-1	/	1	a	−	−	−
63	*L. monocytogenes*	ATCC19114	/	1	b	−	−	−
64	*V. parahemolyticus*	ATCC33847	/	1	b	−	−	−
65	*P. aeruginosa*	ATCC15442	/	1	b	−	−	−
66	*Escherichia coli*	CMCC44103	/	1	c	−	−	−
67	*P. mirabilis*	CMCC49005	/	1	c	−	−	−
68	*S. enteritidis*	CMCC50335	/	1	c	−	−	−
69	*C. sakazakii*	ATCC29544	/	1	b	−	−	−
70	*B. cereus*	ATCC14579	/	1	b	−	−	−
71	*C. jejuni*	ATCC6633	/	1	b	−	−	−

* a, our laboratory isolate. b, ATCC, American Type Culture Collection, USA. c, CMCC, China Medical Culture Collection, China. (+/−) indicate positive and negative signals.

**Table 4:** mPCR and qPCR assay results for natural food samples.

No.	Sample	Test Results for								
		ST7			ST188			ST398		
		Conventional MLST	mPCR	qPCR	Conventional MLST	mPCR	qPCR	Conventional MLST	mPCR	qPCR
1	Milk (*n* = 10)	−	−	−	−	−	−	−	−	−
2		−	−	−	−	−	−	−	−	−
3		−	−	−	−	−	−	−	−	−
4		−	−	−	−	−	−	−	−	−
5		−	−	−	−	−	−	−	−	−
6		−	−	−	−	−	−	−	−	−
7		−	−	−	−	−	−	−	−	−
8		−	−	−	−	−	−	−	−	−
9		−	−	−	−	−	−	−	−	−
10		−	−	−	−	−	−	−	−	−
11	pork meat (*n* = 10)	−	−	−	−	−	−	−	−	−
12		−	−	−	−	−	−	−	−	−
13		−	−	−	−	−	−	−	−	−
14		−	−	−	−	−	−	−	−	−
15		−	−	−	−	−	−	−	−	−
16		−	−	−	−	−	−	−	−	−
17		−	−	−	−	−	−	−	−	−
18		−	−	−	+	+	+	−	−	−
19		−	−	−	−	−	−	−	−	−
20		−	−	−	−	−	−	−	−	−
21	wet rice noodle (*n* = 10)	−	−	−	−	−	−	−	−	−
22		−	−	−	−	−	−	−	−	−
23		−	−	−	−	−	−	−	−	−
24		−	−	−	+	+	+	−	−	−
25		−	−	−	−	−	−	−	−	−
26		−	−	−	−	−	−	−	−	−
27		−	−	−	−	−	−	−	−	−
28		−	−	−	−	−	−	−	−	−
29		−	−	−	−	−	−	−	−	−
30		−	−	−	−	−	−	−	−	−

**Table 5:** Statistical analysis between mPCR/qPCR assays and standard MLST method in actual samples detection.

Species Strain	Number of Samples	Standard MLST Method		mPCR Method		qPCR Method		Sensitivity (%)	Specificity (%)	Efficiency (%)
		+	−	+	−	+	−			
*S. aureus* ST7	30	0	30	0	30	0	30	100	100	100
*S. aureus* ST188	30	2	28	2	28	2	28	100	100	100
*S. aureus* ST398	30	0	30	0	30	0	30	100	100	100

## Data Availability

Data are contained within the article and Supplementary Materials.

## Supplementary Materials

The following supporting information can be downloaded at: https://www.mdpi.com/article/10.3390/molecules30193889/s1, Figure S1: Phylogenetic analysis of *S. aureus*; Figure S2: The specificity evaluation of novel targets for three major *S. aureus* STs by PCR assay; Figure S3: Anti-interference evaluation of primer pairs; Figure S4: Sensitivity of novel target-based PCR assay for detection of three major *S. aureus* STs; Figure S5: Verification of specificity of the reported target for *S. aureus* ST398 by PCR amplification. Table S1: Bacterial strains used for specificity evaluation of mPCR and qPCR methods; Table S2: Bacterial strains whose genomes were used for bioinformatics analysis; Reference [44] is cited in the supplementary materials.
