# Supplementary material for: Novel ST-Specific Molecular Target-Based Method for Simultaneous and Quantitative Detection of Staphylococcus aureus ST7, ST188 and ST398
Source: Molecules. 2025 Sep 26;30(19):3889. doi: 10.3390/molecules30193889 (PMC12526368; doi:10.3390/molecules30193889)
Supplement: Supplementary file 1 [file molecules-30-03889-s001.zip › molecules-3830345-supplementary.pdf]

Supplementary Material

Novel ST-Specific Molecular Target-Based Method for Simultaneous and Quantitative Detection of Staphylococcus aureus ST7, ST188 and ST398

Baoqing Zhou[1, 2, 3†], Xiang Nie[2†], Xudong Mao[1, 2, 4], Jiaxin Chen[2], Jiawen Chen[2], Bingfeng Ma[2], Xin Wu[2,*]

[1]State Key Laboratory of Food Science and Resources, Nanchang University, Nanchang 330047, China

[2]Office of Science and Technology, Jiangxi General Institute of Testing and Certification, Nanchang 330052, China

[3]Nanchang Key Laboratory of Food Rapid Testing, Jiangxi General Institute of Testing and Certification, Nanchang 330200, China

[4]School of Chemistry and Biological Engineering, University of Science and Technology Beijing, Beijing 100083, China

* Correspondence: 18770022505@163.com

† These authors contribute to the manuscript equally.

Table S1. Bacteria strains used for specificity evaluation of mPCR and qPCR method.

No.	Bacterial species	Strains	ST type	Number of strains	Source*	Special target for detection results					
						mPCR			qPCR		
						ST7	ST188	ST398	ST7	ST188	ST398
1	*S. aureus*	192-0, 192-1, 201-0, 201-1, 201-2, 202-1, 203-0, 203-2, 306-1, 322-1	7	10	a	+	-	-	+	-	-
2		3151-1, 3151C1, 3153-1, 3153A1, 3153A2, 3153B3, 3185-1, 3188-1, 3231-1, 3260-1	188	10	a	-	+	-	-	+	-
3		4022C2, 4051A3, 4076A1, 4260A1, 4266C1, 4291A1, 697A, 2816-5, 2816-8, 2831-3	398	10	a	-	-	+	-	-	+
4		16-0	1	1	a	-	-	-	-	-	-
5		4029C2	5	1	a	-	-	-	-	-	-
6		1393	12	1	a	-	-	-	-	-	-
7		52-0	522	1	a	-	-	-	-	-	-
8		24-2	72	1	a	-	-	-	-	-	-
9		1-1	8	1	a	-	-	-	-	-	-
10		223-2	906	1	a	-	-	-	-	-	-
11		3251B1	943	1	a	-	-	-	-	-	-
12		73-1	944	1	a	-	-	-	-	-	-
13		3675C1	950	1	a	-	-	-	-	-	-
14	*S. epidermidis*	612-1	/	1	a	-	-	-	-	-	-
15	*S. hominis*	0651-3	/	1	a	-	-	-	-	-	-
16	*S. haemolyticus*	0770-1	/	1	a	-	-	-	-	-	-
17	*S. capitis*	0640-3	/	1	a	-	-	-	-	-	-
18	*S. warneri*	0629-1	/	1	a	-	-	-	-	-	-
19	*S. saprophyticus*	1045-1	/	1	a	-	-	-	-	-	-
20	*S. sciuri*	0729-7	/	1	a	-	-	-	-	-	-
21	*S. lugdunensis*	0791-2	/	1	a	-	-	-	-	-	-
22	*S. cohnii*	0616-5	/	1	a	-	-	-	-	-	-
23	*S. pasteuri*	0821-1	/	1	a	-	-	-	-	-	-
24	*Shigella sonnei*	0639-1	/	1	a	-	-	-	-	-	-
25	*L. monocytogenes*	ATCC19114	/	1	b	-	-	-	-	-	-
26	*V.parahemolyticus*	ATCC33847	/	1	b	-	-	-	-	-	-
27	*P. aeruginosa*	ATCC15442	/	1	b	-	-	-	-	-	-
28	*E. coli*	CMCC44103	/	1	c	-	-	-	-	-	-
29	*P. mirabilis*	CMCC49005	/	1	c	-	-	-	-	-	-
30	*S. enteritidis*	CMCC50335	/	1	c	-	-	-	-	-	-
31	*C. sakazakii*	ATCC29544	/	1	b	-	-	-	-	-	-
32	*B. cereus*	ATCC14579	/	1	b	-	-	-	-	-	-
33	*C. jejuni*	ATCC6633	/	1	b	-	-	-	-	-	-

* a, our laboratory; b, Guangdong Huankai Co., Ltd., China

b, ATCC, American Type Culture Collection, USA.

c, CMCC, China Medical Culture Collection, China.

Result (+/-) indicate positive and negative signals.

Table S2. Bacterial strains which genomes were used for bioinformatics analysis [44].

Species	STs	Amount	Assembly
Staphylococcus aureus	ST1	13	GCA_000011265.1, GCA_000011525.1, GCA_001018645.2, GCA_001019125.2, GCA_001019535.2, GCA_001879545.1, GCA_002209325.1, GCA_002633825.1, GCA_002795285.1, GCA_003573835.1, GCA_003952625.1, GCA_003957315.1, GCA_900620215.1
	ST105	4	GCA_000016805.1, GCA_000017125.1, GCA_000969225.1, GCA_003330905.1
	ST1093	1	GCA_001307235.1
	ST1148	1	GCA_900474555.1
	ST121	6	GCA_001018845.2, GCA_001444345.1, GCA_003354545.1, GCA_003354605.1, GCA_003354645.1, GCA_003354905.1
	ST133	6	GCA_000210315.1, GCA_003490125.1, GCA_003955945.1, GCA_900635315.1, GCA_900636335.1, GCA_900636695.1
	ST15	9	GCA_001611325.1, GCA_001611345.1, GCA_001611405.1, GCA_001611425.1, GCA_003354865.1, GCA_003354925.1, GCA_900323905.1, GCA_900324045.1, GCA_900324065.1
	ST1516	1	GCA_004136235.1
	ST152	2	GCA_002803885.1, GCA_900004855.1
	ST1750	1	GCA_003193685.1
	ST188	3	GCA_002140115.1, GCA_003010875.1, GCA_003573855.1
	ST22	10	GCA_000284535.1, GCA_000695215.1, GCA_001018835.2, GCA_002786535.1, GCA_003184985.1, GCA_003185005.1, GCA_004153365.1, GCA_900620235.1, GCA_900635245.1, GCA_900635905.1
	ST225	1	GCA_000025145.2
	ST228	13	GCA_000382965.1, GCA_000382985.1, GCA_000383005.1, GCA_000967325.1, GCA_000967345.1, GCA_000967365.1, GCA_000967385.1, GCA_000967405.1, GCA_003354565.1, GCA_003354585.1, GCA_003354705.1, GCA_003354765.1, GCA_003354985.1
	ST2389	2	GCA_003945405.1, GCA_003945425.1
	ST239	25	GCA_000027045.1, GCA_000145595.1, GCA_000204665.1, GCA_000418345.1, GCA_000485885.1, GCA_000709475.1, GCA_001515665.1, GCA_001515685.1, GCA_001515705.1, GCA_001515745.1, GCA_001515765.1, GCA_001641025.1, GCA_002895385.1, GCA_003264815.1, GCA_003394105.1, GCA_004136255.1, GCA_900475055.1, GCA_900607245.1, GCA_900607255.1, GCA_900607265.1, GCA_900607275.1, GCA_900607285.1, GCA_900607295.1, GCA_900607305.1, GCA_900620245.1
	ST240	1	GCA_900474575.1
	ST241	1	GCA_003264775.1
	ST243	3	GCA_000756205.1, GCA_002202075.1, GCA_002202095.1
	ST2454	2	GCA_002850415.1, GCA_002952015.1
	ST2490	1	GCA_000189455.3
	ST25	3	GCA_001611365.1, GCA_001611385.1, GCA_900635095.1
	ST250	2	GCA_000012045.1, GCA_000626615.3
	ST254	7	GCA_000010465.1, GCA_002310395.1, GCA_002310435.1, GCA_003354665.1, GCA_003354945.1, GCA_003354965.1, GCA_900092595.1
	ST2544	1	GCA_002850395.1
	ST27	1	GCA_001027045.1
	ST30	16	GCA_000160335.2, GCA_000772025.1, GCA_000953255.1, GCA_001018975.2, GCA_001019395.2, GCA_002633785.1, GCA_003031425.1, GCA_003193745.1, GCA_003354625.1, GCA_003354685.1, GCA_003354725.1, GCA_003354745.1, GCA_003354785.1, GCA_003827915.1, GCA_900635335.1, GCA_900637155.1
	ST338	1	GCA_001456215.1
	ST36	2	GCA_000011505.1, GCA_900478245.1
	ST398	34	GCA_000009585.1, GCA_000296595.1, GCA_001465635.1, GCA_001465675.1, GCA_001465755.1, GCA_001887075.1, GCA_002025125.1, GCA_002089035.2,

			GCA_002089055.1, GCA_002089075.2, GCA_002089095.2, GCA_002089115.2, GCA_002204575.1, GCA_003029645.1, GCA_003111745.1, GCA_900324205.1, GCA_900324215.1, GCA_900324225.1, GCA_900324235.1, GCA_900324255.1, GCA_900324265.1, GCA_900324275.1, GCA_900324285.1, GCA_900324295.1, GCA_900324305.1, GCA_900324315.1, GCA_900324325.1, GCA_900324335.1, GCA_900324345.1, GCA_900324355.1, GCA_900324365.1, GCA_900324385.1, GCA_900324405.1, GCA_900324415.1
	ST425	2	GCA_000237265.1, GCA_900635255.1
	ST4307	1	GCA_003030085.1
	ST433	2	GCA_001956755.1, GCA_003030065.1
	ST45	5	GCA_000412775.1, GCA_001594205.1, GCA_002633765.1, GCA_003073635.1, GCA_003073755.1
	ST4618	1	GCA_000159535.2
	ST464	6	GCA_001548295.1, GCA_002025145.1, GCA_003827735.1, GCA_003827835.1, GCA_900635305.1, GCA_900635505.1
	ST49	1	GCA_000452385.2
	ST5	45	GCA_000009645.1, GCA_000009665.1, GCA_000010445.1, GCA_000024585.1, GCA_000253135.1, GCA_000597965.1, GCA_001018775.2, GCA_001019205.2, GCA_001019255.2, GCA_001019305.2, GCA_001019435.2, GCA_001019495.2, GCA_001019575.2, GCA_001278745.1, GCA_001281145.1, GCA_001548415.1, GCA_001618305.1, GCA_001640885.1, GCA_001640905.1, GCA_001640925.1, GCA_001717665.2, GCA_001725965.1, GCA_001975005.1, GCA_001975045.1, GCA_002204555.1, GCA_002633805.1, GCA_002633865.1, GCA_003031485.1, GCA_003073655.1, GCA_003073715.1, GCA_003073775.1, GCA_003193705.1, GCA_003193885.1, GCA_003194005.1, GCA_003194025.1, GCA_003194405.1, GCA_003203615.1, GCA_003203635.1, GCA_003203655.1, GCA_003203675.1, GCA_003351925.1, GCA_003991015.1, GCA_004208595.1, GCA_900017775.1, GCA_900620225.1
	ST50	3	GCA_000462955.1, GCA_003609895.1, GCA_003609915.1
	ST507	1	GCA_001019485.2
	ST508	1	GCA_003193765.1
	ST582	1	GCA_001656045.1
	ST59	6	GCA_000237125.1, GCA_000470845.1, GCA_000470865.1, GCA_000737615.1, GCA_002442975.1, GCA_002633845.1
	ST612	1	GCA_003111725.1
	ST632	1	GCA_003431365.1
	ST7	1	GCA_001656075.1
	ST700	2	GCA_000189435.3, GCA_003627835.1
	ST71	1	GCA_003186125.1
	ST72	10	GCA_000463055.1, GCA_001549655.1, GCA_001549675.1, GCA_001580495.1, GCA_002214665.1, GCA_003193665.1, GCA_003193785.1, GCA_003342735.1, GCA_003342775.1, GCA_003347055.1
	ST772	2	GCA_000828035.1, GCA_004153345.1
	S8	87	GCA_000013425.1, GCA_000013465.1, GCA_000017085.1, GCA_000245495.1, GCA_000568455.1, GCA_000695875.1, GCA_000746505.1, GCA_000815045.1, GCA_000815085.1, GCA_000815125.1, GCA_000815165.1, GCA_000815205.1, GCA_000815245.1, GCA_001018655.2, GCA_001018685.2, GCA_001018725.2, GCA_001018735.2, GCA_001018805.2, GCA_001018915.2, GCA_001019275.2, GCA_001019415.2, GCA_001021875.1, GCA_001021895.1, GCA_001027105.1, GCA_001045795.2, GCA_001045995.2, GCA_001183705.3, GCA_001183725.2, GCA_001580515.1, GCA_001717645.3, GCA_001717685.3, GCA_001717705.2, GCA_001717725.2, GCA_001717975.3, GCA_001735655.2, GCA_001900185.1, GCA_001956815.1, GCA_002000565.1, GCA_002000585.1, GCA_002000605.1, GCA_002000625.1, GCA_002000645.1, GCA_002000665.1, GCA_002000685.1, GCA_002000705.1, GCA_002000725.1, GCA_002000745.1, GCA_002000765.1, GCA_002000785.1, GCA_002000805.1, GCA_002000825.1, GCA_002000845.1, GCA_002000865.1, GCA_002085525.1, GCA_002088995.1, GCA_002355355.1,

			GCA_002356675.1, GCA_002786465.1, GCA_003073395.1, GCA_003073415.1, GCA_003073435.1, GCA_003193725.1, GCA_003193965.1, GCA_003203595.1, GCA_003354805.1, GCA_003354885.1, GCA_003425835.1, GCA_003425885.1, GCA_003426145.1, GCA_003595365.1, GCA_003595385.1, GCA_003595405.1, GCA_003595425.1, GCA_003595445.1, GCA_003595465.1, GCA_003595485.1, GCA_003595505.1, GCA_004118995.1, GCA_004193875.1, GCA_004193895.1, GCA_900129335.1, GCA_900474525.1, GCA_900474725.1, GCA_900474735.1, GCA_900475245.1, GCA_900620255.1, GCA_900635265.1
	ST80	3	GCA_000239235.1, GCA_001296985.1, GCA_001457495.1
	ST87	1	GCA_003288395.1
	ST88	2	GCA_001986135.1, GCA_900096745.1
	ST9	6	GCA_001298325.2, GCA_003030225.1, GCA_003432345.1, GCA_003432365.1, GCA_004136655.1, GCA_900474715.1
	ST923	1	GCA_001046095.2
	ST93	1	GCA_000144955.1
	ST97	4	GCA_003186105.1, GCA_900474665.1, GCA_900474755.1, GCA_900635285.1
Non-target *S.aureus*	/	27	GCA_000009005.1, GCA_001558795.2, GCA_001641045.1, GCA_001880265.1, GCA_002097595.2, GCA_002386245.1, GCA_003010475.1, GCA_000769575.1, GCA_003425545.1, GCA_003425915.1, GCA_003609855.1, GCA_003855575.1, GCA_003944865.1, GCA_004026165.1, GCA_004026185.1, GCA_900155335.1, GCA_900323925.1, GCA_900323955.1, GCA_900323965.1, GCA_900324245.1, GCA_900474535.1, GCA_900474565.1, GCA_900474675.1, GCA_900474695.1, GCA_900635275.1, GCA_900636355.1, GCA_900636395.1
S. epidermidis	/	21	GCA_000007645.1, GCA_000011925.1, GCA_000751035.1, GCA_000759555.1, GCA_000934225.1, GCA_001956655.2, GCA_002085695.1, GCA_002215535.1, GCA_002749455.1, GCA_002749515.1, GCA_002850315.1, GCA_002944995.1, GCA_002954055.1, GCA_003119275.1, GCA_003325735.1, GCA_003812425.1, GCA_003856395.1, GCA_003856455.1, GCA_900086615.1, GCA_900636255.1, GCA_900638695.1
S. haemolyticus	/	8	GCA_000009865.1, GCA_000972725.1, GCA_001611955.1, GCA_002906595.1, GCA_002906615.1, GCA_002952715.2, GCA_003956005.1, GCA_900240195.1
S. argenteus	/	4	GCA_000236925.1, GCA_001891145.1, GCA_002812345.2, GCA_003595345.1
S. auricularis	/	1	GCA_900478415.1
S. felis	/	1	GCA_003012915.1
S. hyicus	/	2	GCA_000816085.1, GCA_900474585.1
S. cohnii	/	2	GCA_001990205.1, GCA_002984565.1
S. condimenti	/	2	GCA_001618885.1, GCA_001922405.1
S. equorum	/	3	GCA_001432245.1, GCA_001682235.1, GCA_001683315.1
S. pasteuri	/	2	GCA_000494875.1, GCA_002442915.1
S. pettenkoferi	/	1	GCA_002208805.2
S. kloosii	/	1	GCA_003019255.1
S. lugdunensis	/	10	GCA_000025085.1, GCA_000270465.1, GCA_001558775.1, GCA_001558815.2, GCA_002073395.2, GCA_002250095.2, GCA_002407165.1, GCA_002591215.1, GCA_900474705.1, GCA_900478255.1
S. lutrae	/	1	GCA_002101335.1
S. stepanovicii	/	1	GCA_900187075.1
S. hominis	/	2	GCA_002850375.1, GCA_003812505.1
S. capitis	/	3	GCA_001028645.1, GCA_002356175.1, GCA_002591175.1
S. carnosus	/	2	GCA_000009405.1, GCA_001701005.1
S. pseudintermedius	/	6	GCA_000185885.1, GCA_000189495.1, GCA_001682335.1, GCA_001682435.2, GCA_001685665.2, GCA_003627735.1
S. saprophyticus	/	6	GCA_000010125.1, GCA_001558275.2, GCA_001558375.2, GCA_002208905.2, GCA_002209265.2, GCA_900240075.1
S. schleiferi	/	5	GCA_001188855.1, GCA_001188875.1, GCA_001188895.1, GCA_001188915.1, GCA_001548255.1
S. sciuri	/	3	GCA_002072755.1, GCA_002209165.2, GCA_900474615.1
S. simiae	/	1	GCA_900187055.1

S. warneri	/	3	GCA_000332735.1, GCA_003571725.1, GCA_003691405.1
S. xylosus	/	4	GCA_000706685.1, GCA_000709415.1, GCA_000953575.1, GCA_002078255.1
S. muscae	/	2	GCA_003019275.1, GCA_900187005.1
S. nepalensis	/	3	GCA_002442895.1, GCA_002442935.1, GCA_003097555.1
S. simulans	/	7	GCA_001559115.2, GCA_002386185.1, GCA_003006055.1, GCA_003006075.1, GCA_003076375.1, GCA_003096155.1, GCA_900474685.1
total		505	

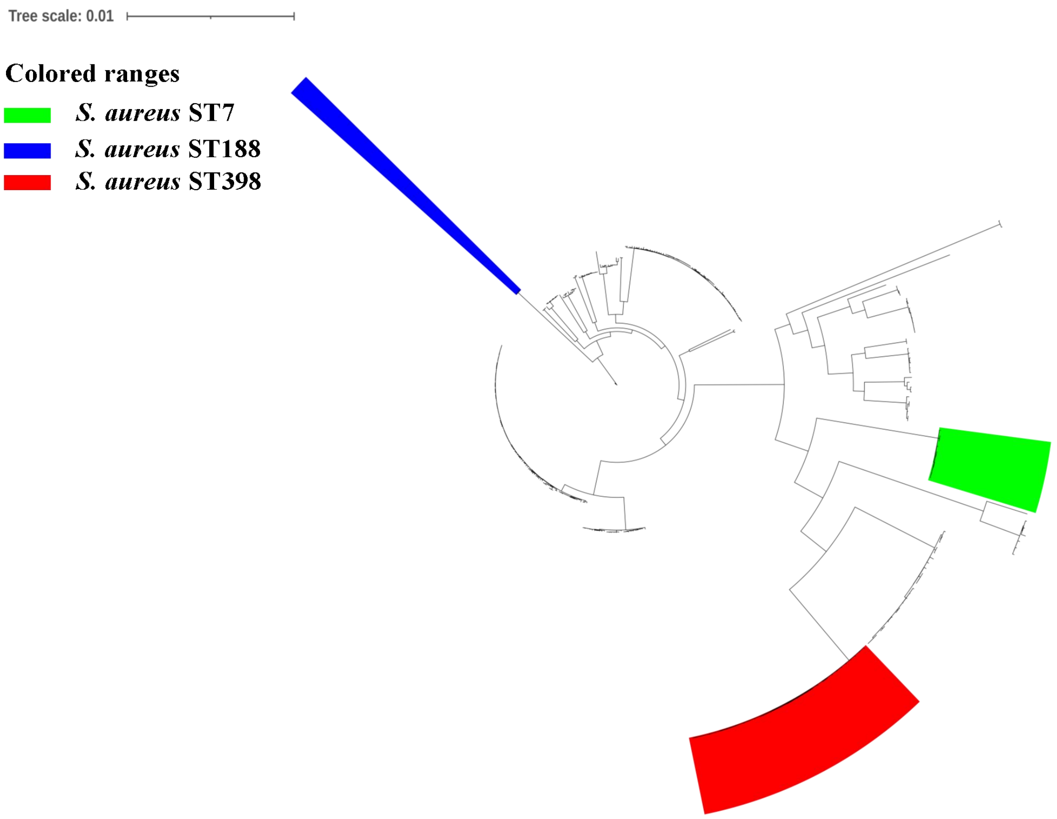

Figure S1. Phylogenetic analysis of *S. aureus*.

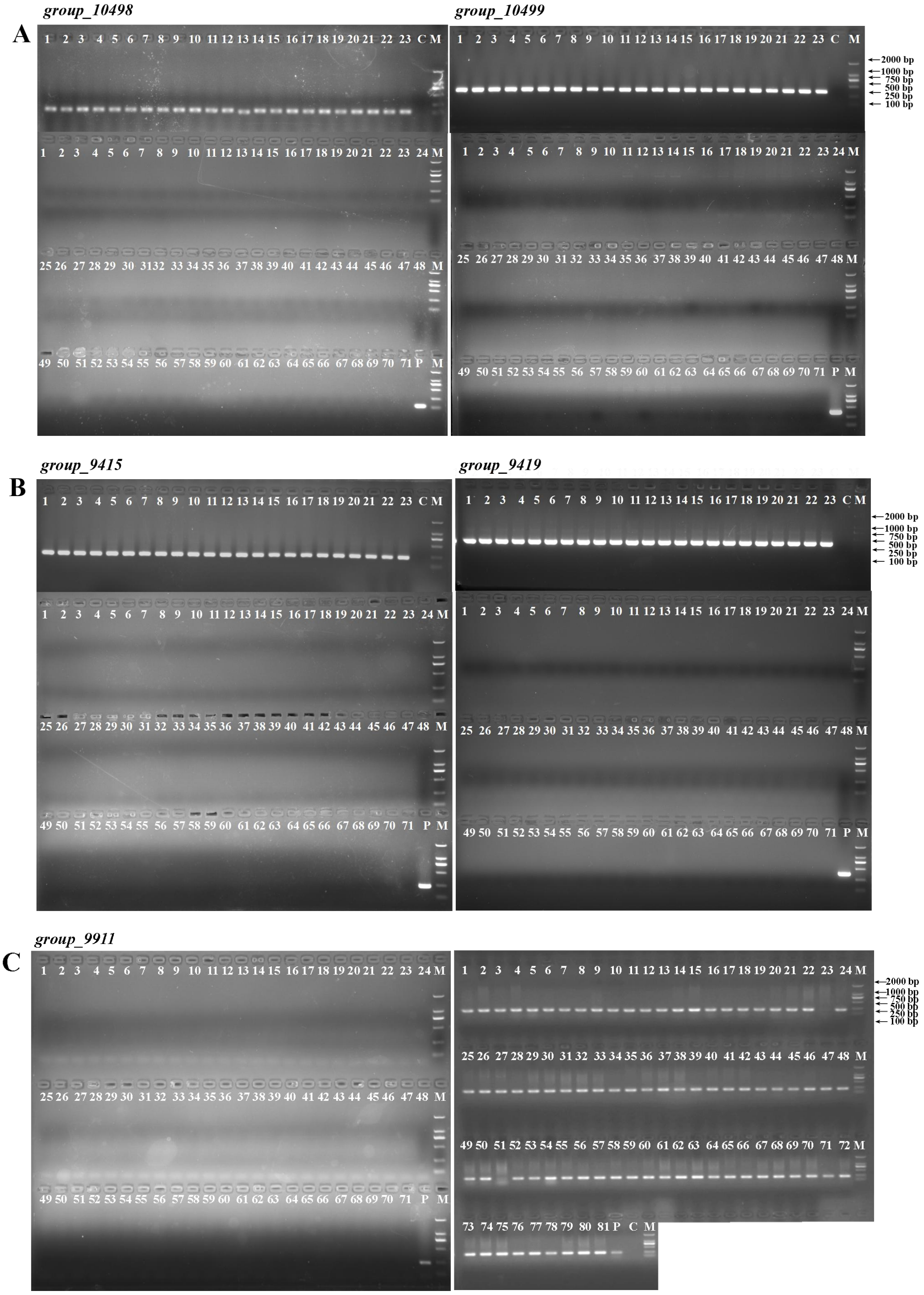

Figure S2. The specificity evaluation of novel targets for three major *S. aureus* STs by PCR assay. (A) PCR results of novel molecular detection gene (*group_10498* and *group_10499*) specific for *S. aureus* ST7; lanes 1-23 (above): target *S. aureus* ST7 strains, lanes 1-71 (below): other non-target *S. aureus* STs strains; (B) PCR results of novel molecular detection gene (*group_9415* and *group_9419*) specific for *S. aureus* ST188, lanes 1-23 (above): target *S. aureus* ST188 strains, lanes 1-71 (below): other

non-target *S. aureus* STs strains; (C) PCR results of novel molecular detection gene (*group_9911*) specific for *S. aureus* ST398, lanes 1-71 (above): other non-target *S. aureus* STs strains, lanes 1-81 (below): target *S. aureus* ST398 strains; all lane M: DL2000 DNA marker, P: positive control, C:negative control.

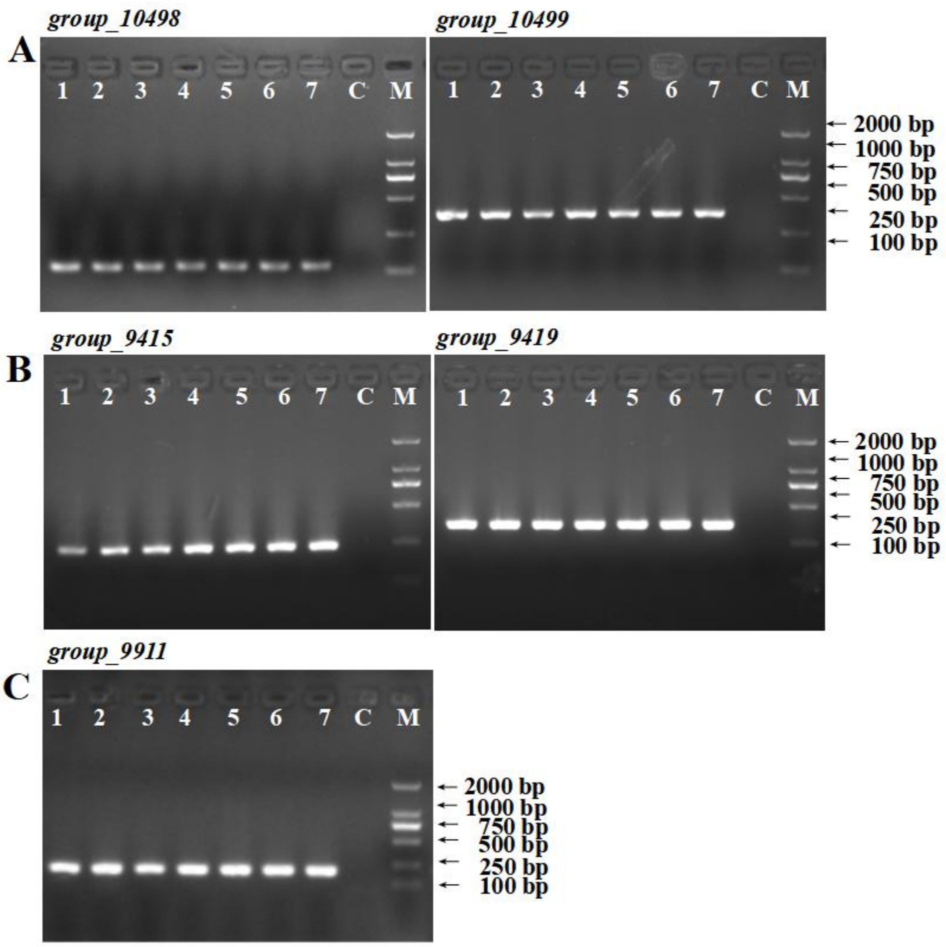

Figures S3. Anti-interference evaluation of primer pairs. Primer pairs from *group_10498* and *group_10499* for *S. aureus* ST7 detection (A), primer pairs from *group_9415* and *group_9419* for *S. aureus* ST188 detection (B) and primer pairs from *group_9911* for *S. aureus* ST398 detection (C), respectively; lane M: DL2000 DNA marker, lanes 1-7: the other *S. aureus* ST8 1-1 of 10^1 ~ 10^7 CFU/mL mixed three major *S. aureus* STs (initial concentration of 10^6 CFU/mL), respectively for PCR detection; lane C: negative control.

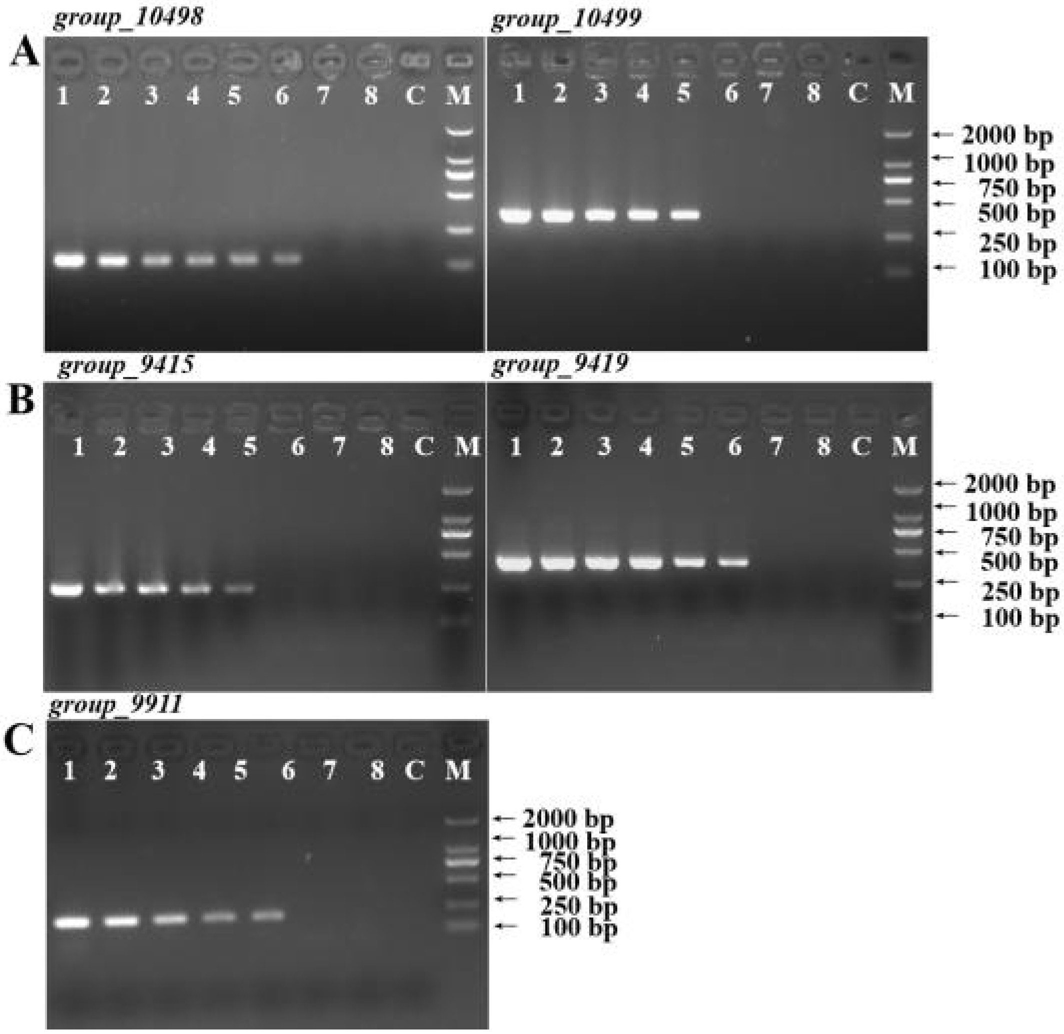

Figures S4. Sensitivity of novel targets based-PCR assay for detection of three major *S. aureus* STs. Limits of detection primer sets for the genes: *group_10498* and *group_10499* of *S. aureus* ST7 (A), *group_9415* and *group_9419* of *S. aureus* ST188 (B) and *group_9911* of *S. aureus* ST398 (C), respectively; lane M: DL2000 DNA marker, lanes 1-7: bacterial culture concentrations per PCR assay: 10^8-10^1 CFU/mL, lane C: negative control.

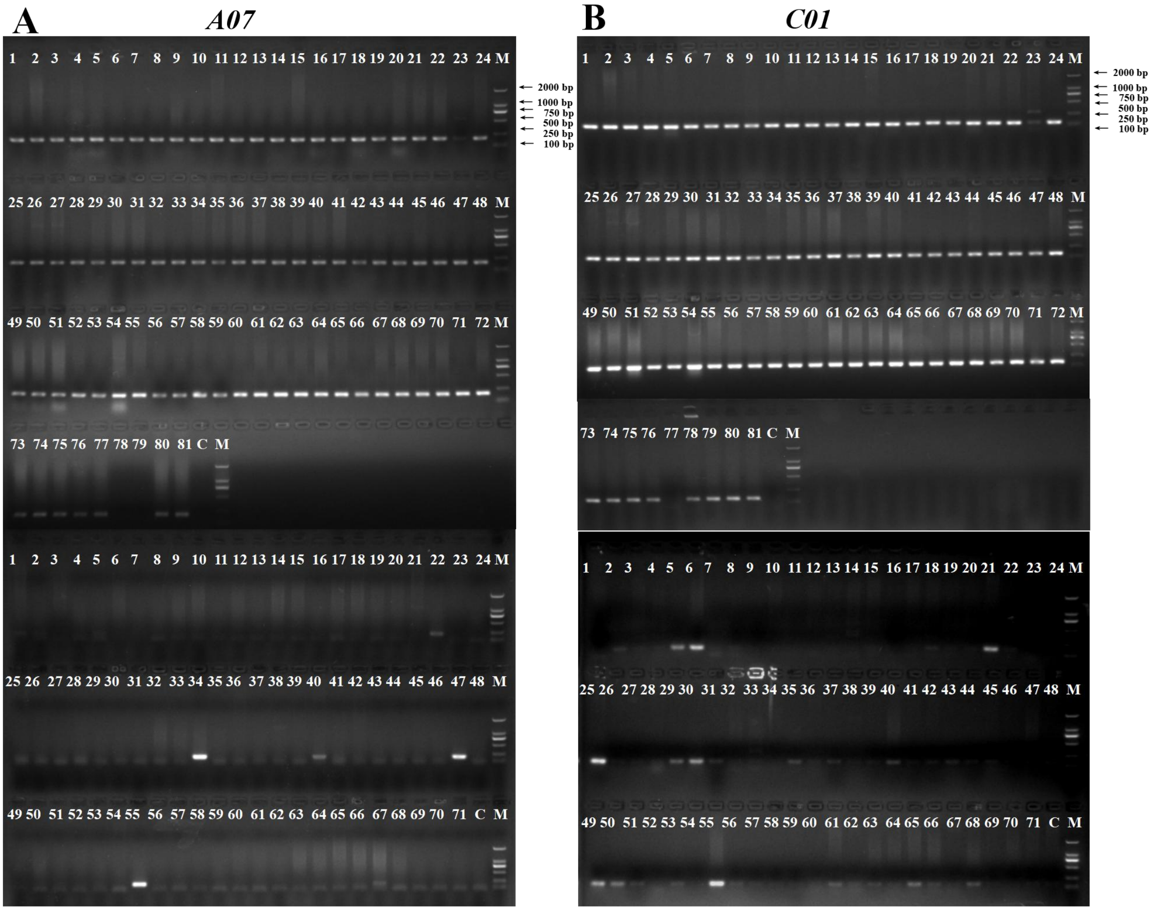

Figures S5. Verification of specificity of the reported target for *S. aureus* ST398 by PCR amplification. (A) Evaluation the specificity of target *A07* (A) and *C01* (B) for detection of *S. aureus* ST398; lanes 1-81 (above): target *S. aureus* ST398 strains, lanes 1-71 (below): other non-target *S. aureus* ST398 strains, lane M: DL2000 DNA marker, lane C: negative control.
